# Shprintzen-Goldberg syndrome: a rare disorder

**DOI:** 10.11604/pamj.2016.23.227.7482

**Published:** 2016-04-25

**Authors:** Sankalp Yadav, Gautam Rawal

**Affiliations:** 1 General Duty Medical Officer-II, Chest Clinic Moti Nagar, North Delhi Municipal Corporation, New Delhi, India; 2 Critical Care Department, Rockland Hospital, Qutab Institutional Area, New Delhi, India

**Keywords:** Craniosynostosis, Marfanoid features, Shprintzen-Goldberg syndrome

## Abstract

Shprintzen-Goldberg Syndrome is an extremely infrequent disorder of connective tissue, characterized by craniosynostosis and marfanoid features, also known as Marfanoid Craniosynostosis syndrome. The syndrome was first introduced by Sugarman and Vogel’ (1981) however, Shprintzen and Goldberg established this as a separate clinical entity in the year 1982. Since then, approximately sixty such cases have been set down in writing in the medical literature. Herein, we present a short review of literature of this rare connective disorder, in order to create awareness about this condition, as the magnitude of this disorder is not measured properly due to the paucity of literature.

## Commentary

Shprintzen-Goldberg Syndrome (SGS) is an extremely sparse group of disorders characterized by craniosynostosis with marfanoid features and characteristic facies [1–3]. The phenotype of this syndrome is variable because it combines craniofacial, neurological, skeletal, cardiovascular and other connective tissue abnormalities. It is a well-documented syndrome with craniosynostosis, mental retardation, skeletal anomalies with marfanoid habitus, Hirschsprung disease, and aortic root dilatation [3–6]. Furthermore, the craniofacial abnormalities, most frequently described in the SGS include dolichocephaly, low-set ears, a high prominent forehead, proptosis, hypertelorism, divergent strabismus, down slanting eyes, high arched narrow palate, and maxillary hypoplasia [1, 2]. The main skeletal findings in SGS are arachnodactyly, flat feet, pectus deformity, scoliosis and hypermobile joints [1]. Prevalence of SGS is unknown, and it is difficult to determine the number of affected individuals, because some cases diagnosed as SGS could well be Marfan syndrome or Loeys-Dietz syndrome, which is the result of considerable phenotypic overlapping of SGS with both Marfan syndrome or Loeys-Dietz syndrome [1, 3, 7–9]. Both males and females are affected. Differential diagnosis included Marfan syndrome, Loeys-Dietz syndrome, Idaho syndrome-II, Antley-Bixler syndrome, congenital contractural arachnodactyly and several other craniosynostotic syndromes. SGS almost always results from new (de novo) gene mutations and occurs in people with no history of the disorder in their family. Very rarely, cases with this disorder have inherited the altered gene from a normal parent who has a gene mutation only in their egg or sperm cells. This type of mutation which is present only in reproductive cells is known as germline mosaicism. Besides mutations, other factors like environmental factors or a combination of both; also play a major role in the development of SGS. SGS shares with Marfan syndrome, as it is also caused by mutation in the Fibrillin-1 gene (FBN1) located on 15q21.1, the same gene responsible for Marfan syndrome [3–5]. Similar to Marfan syndrome, it is inherited in an autosomal dominant fashion or sporadic mutation; however X linked recessive inheritance could not be excluded [10, 11]. Common features of Marfan syndrome and SGS includes skeletal anomalies such as arachnodactyly, scoliosis and marked dilatation of the aorta at the root [5]. Craniosynostosis, delayed development renal anomalies, and mild to moderate intellectual disability are additional features of SGS [1, 5]. Besides, heart abnormalities are comparatively more common and usually more severe in Marfan syndrome [7]. Other common features of SGS include heart or brain abnormalities, hypotonia in infancy, and an abdominal wall abnormality leading to soft out-pouching around the belly button (umbilical hernia) or lower abdomen (inguinal hernia) [7]. SGS also shows signs and symptoms similar to Loeys-Dietz syndrome; however intellectual disability is more likely to occur in SGS than in Loeys-Dietz syndrome. In addition, heart abnormalities are more common and usually more severe in Loeys-Dietz syndrome [7]. SGS is also reported to share similarities with Frontometaphyseal dysplasia and Melnick-Needles syndrome, two disorders in the otopalatodigital spectrum disorders, with findings like bowed tibiae, tall strature, square-shaped vertebrae, and occasionally, fusion of upper cervical vertebrae. However; the SGS is distinguished from both these by presence of intellectual disability and craniosynostosis [12].

There are also a number of different syndromes associated with both craniosynostosis and marfanoid body type. SGS must be differentiated from these syndromes. Two such syndromes include Idaho syndrome-II and Antley-Bixler syndrome. Idaho syndrome-II has comparatively less severe craniofacial problems than SGS and has abnormal leg bones and absent patellae [13]. Antley-Bixler syndrome is an inherited syndrome with craniofacial abnormalities, abnormal arm and leg bones, and fractures in the femur [14]. These characteristics are different from SGS. It was also different from congenital contractural arachnodactyly (CCA) due to the presence of cardiovascular and ocular complications which are absent in the CCA. Greally et al. 1998, suggested a wide range of dysmorphic features associated with SGS [1]. The patient may present with the craniofacial features like proptosis, myopia, telecanthus, strabismus, hypertelorism, down slanting palpebral fissures, micrognathia and/or retrognathia, high narrow palate with prominent palatine ridges, malar flattening/hypoplasia, dolichocephaly with or without scaphocephaly; tall or prominent forehead, and low-set and posteriorly rotated ear in SGS [1, 4, 12, 15]. The patient may also have abnormal neurological, skeletal, cardiovascular, genitourinary features [12, 16, 17]. Besides, a number of other features like hernias and abdominal wall defects, loss of subcutaneous fat, arterial tortuosity with or without aneurysms, cleft palate, broad/bifid uvula, dural ectasia may also be present [12]. The SGS is caused by mutations in a gene that is involved in the formation of connective tissue, it has been traced to a defect of the chromosome 15 [4, 5]. Germline mosaicism has been suggested in SGS, mutations in three genomic loci have been linked to SGS; thus SGS is a molecularly heterogeneous disorder [18]. This also emphasizes that there may be multiple other genetic factors, that result in a common clinical phenotype. Many investigators have linked a fourth region (15q25-qter) in the etiology of this disorder [18].

Previous reports have described that mutations in the fibrillin-1 (FBN1) gene, on chromosome 15q21.1, have been found to result in Marfan syndrome, a dominantly inherited disorder characterized by clinically skeletal, ocular and cardiovascular abnormalities [19–22]. Mutations of FBN1 are also seen in SGS [4, 20]. In a relatively recent report describing a Japanese boy with clinical findings consistent with SGS, Kosaki et al. 2006, identified a 3662E-A transition (134797.0045) leading to cys 1221-to-tyr (C 1221 Y) substitution in the FBN 1 gene [23]. However, Greally 2006, mentioned that the mutations in the SKI gene are the only cause for the SGS and are inherited in autosomal dominant manner [7, 8, 12, 24]. Mutations in the genes for transforming growth factor-beta receptor (TGFBR) have been identified in patients with Marfan syndrome and Marfan-like connective tissue disorders, like Loeys-Dietz syndrome, Marfan Syndrome type-2, Furlong syndrome, Camurati-Engelmann syndrome and SGS [25, 26]. However, not all cases are results of mutation in either FBN1 or TGFBR or SKI gene, as other genes may also be involved in this condition, as sometimes, the underlying genetic cause could not be identified and thus further research is warranted for a better understanding of the disease pathogenesis [18]. The case of SGS, may serve as a clue that many patients with marfanoid phenotypes and dysmorphic features may have grave underlying cardiac conditions. All such cases should be thoroughly examined for underlying cardiovascular abnormalities. SGS is as such, not a life threatening disorder; but complications due to mental retardation and respiratory difficulty can cause problems. Besides, prognosis is poor in cases having cardiac complications, if not detected early.

Cardiovascular complications are the most common immediate cause of death because of progressive aortic root dilation, mitral valve prolapse and mitral regurgitation. Early detection of these defects by echocardiography and consultation with a cardiologist is required. Aortic root must be evaluated and measured routinely to minimize the risk of rupture. Enlarged aortic roots may need to be surgically repaired [12]. The regular Ophthalmological examination must be done in order to prevent retinal detachment and other ophthalmological problems. Craniofacial problems or pectus sometimes require surgical correction. In patients with hydrocephalus, shunting (surgical placement of a shunt to drain the collected fluid in the brain into the abdominal cavity to relieve pressure) may be required. Orthopaedic devices may be required for scoliosis or other bone abnormalities [12]. The physiotherapy should be provided to ease the joint contractures [12]. Special education for mentally retarded individuals or individuals with developmental delay is recommended. Besides genetic counselling of all such individuals and their families should always be done. The secondary complications like subacute bacterial endocarditis should be taken into account and appropriate prophylaxis should be provided during dental procedures or other similar works which may contaminate the bloodstream with bacteria [12].

In conclusion, SGS is a rare connective tissue disorder with marfanoid features. The patient may present to the surgeon with abdominal wall defect such as an umbilical or inguinal hernia. A high index of suspicion is required to rule out associated cardiovascular anomalies to prevent potential complications in the perioperative and postoperative period [27]. A systematic approach to the patients involving a physician, pediatrician, ophthalmologist, cardiologist, clinical geneticist, otorhinolaryngologist, speech and language pathologist, radiologist, physiotherapist and a surgeon are a must for proper management of such cases.
